# The use of intraoperative tractography in brain tumor and epilepsy surgery: a systematic review and meta-analysis

**DOI:** 10.3389/fnimg.2025.1563996

**Published:** 2025-06-17

**Authors:** Holly Aylmore, Fiona Young, Kristian Aquilina, Chris A. Clark, Jonathan D. Clayden

**Affiliations:** ^1^Developmental Neurosciences Research and Teaching Department, UCL Great Ormond Street Institute of Child Health, University College London, London, United Kingdom; ^2^Department of Medical Physics and Biomedical Engineering, University College London, London, United Kingdom; ^3^Department of Neurosurgery, Great Ormond Street Hospital for Children, London, United Kingdom

**Keywords:** diffusion tensor imaging, tractography, brain tumors, epilepsy, neurosurgery

## Abstract

**Introduction:**

Tractography is the only available technique for visualizing whitematter pathways within the living brain. Avoiding these pathways during surgical interventions for brain tumors and epilepsy is key to reducing postoperative neurological deficits whilst achieving maximum safe resection. Despite this, the use of intraoperative tractography is not widely adopted in clinical practice, with time required to run analyses often cited as a limitation. This systematic review and meta-analysis aimed to assess the impact of intraoperative tractography on neurosurgical outcomes in both tumor and epilepsy surgeries.

**Methods:**

Conducted in accordance with PRISMA guidelines, five major databases were searched using neurosurgery, tractography, brain tumor, and epilepsy terms. Original primary research studies in English were included. A risk of bias analysis was conducted using the MINORS tool.

**Results:**

The search strategy identified 2,611 papers. Following de-duplication and screening, 26 papers were included in the final analysis. Risk of bias was found to be moderate. Findings suggest that the use of intraoperative tractography has the potential to improve surgical outcomes for patients undergoing tumor and epilepsy surgery. Meta-analysis indicated a good rate of gross total resection, 79%, and only three studies of brain tumors and one study of epilepsy reported worsening of neurological deficits.

**Discussion:**

Though the evidence supporting its use remains limited, results indicate that intraoperative tractography can be a valuable tool in improving neurosurgical outcomes and reducing the risk of postoperative deficits. Further research is required to determine optimal use in clinical practice.

**Systematic review registration:**

https://www.crd.york.ac.uk/PROSPERO/view/CRD42023427427, Identifier: CRD42023427427.

## 1 Introduction

The risks of brain tumor and epilepsy surgery have significantly decreased in recent decades in part due to remarkable progress in medical technology (Hall et al., 2000; Johnson and Stacey, 2008; Wirtz et al., 2000). Rapid improvements in imaging quality and methodology have allowed improved visualization of the brain, aiding the development of more precise and accurate surgical interventions. The use of intraoperative magnetic resonance imaging (iMRI) in particular allows for real-time imaging during surgery and enabling surgeons to make informed decisions on how to proceed.

Diffusion tensor imaging (DTI) allows for the visualization of white matter (WM) tracts and their orientations (Basser et al., 1994; Basser and Pierpaoli, 1996). Within tissues diffusion can be free, hindered, or restricted (Bihan, 1995) and categorized as anisotropic or isotropic. Anisotropic diffusion describes situations in which the diffusion of water molecules is not uniform in all directions, and so the diffusion coefficient varies depending on the direction in which it is measured. This commonly occurs when barriers and microstructures within the tissue impede or influence the movement of water molecules.

Three-dimensional maps of WM tracts can be generated using tractography, in which directional information can be used to track the route of axonal bundles from voxel to voxel indicating the approximate underlying structure of the WM tract (Basser et al., 2000). It is important to note that tractography does not directly trace individual WM fibers but instead illustrates the path of least resistance to water diffusion. Given the complexity of these tracts, the generated map should not be regarded as a complete representation of axons but an estimation of their location (Jeurissen et al., 2019).

**Figure 1:**
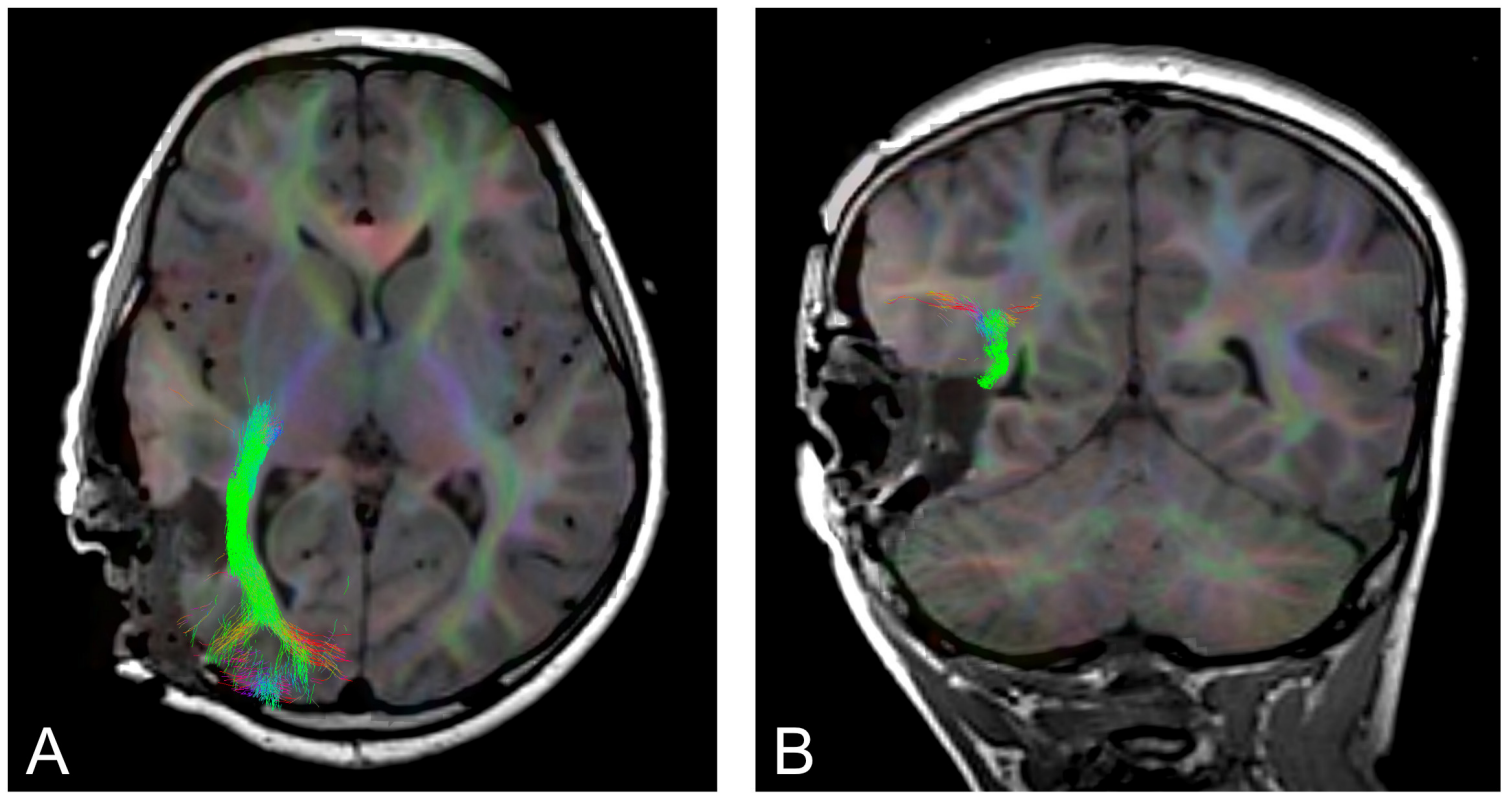
An example of intraoperative tractography of the optic radiation, post tumor resection. **(A)** axial slice, **(B)** coronal slice.

Brain tumors can displace, infiltrate, and reorganize WM tracts, disrupting structural organization and resulting in decreased anisotropy (Duffau, 2005; Witwer et al., 2002). Once surgery has begun and tissue begins to be resected, the surrounding tissue deforms and moves, a phenomenon known as brain shift, which means that pre-surgical images are no longer accurate representations of WM pathways (Nimsky, 2010). Epilepsy surgery is also subject to brain shift (Yang et al., 2017). The use of tractography intraoperatively (Figure 1) allows surgeons to map WM tracts and adjust their approach in real time permitting more precise delineation of surgical margins, preserving critical neural pathways, and minimizing the risk of postoperative neurological deficits (Nimsky et al., 2001; do Amaral et al., 2021).

Maximizing extent of resection (EOR) of tumors is linked to overall survival (Berger and Rostomily, 1997; Sanai and Berger, 2008; Laws et al., 2003; Lacroix et al., 2001; Oppenlander et al., 2014; Scherer et al., 2020). Numerous studies have consistently demonstrated that achieving maximum safe resection of brain tumors yields substantial prognostic advantages and as a result it has become the cornerstone of managing both low and high grade brain tumors (Krivosheya and Prabhu, 2017; Rao, 2017; Aghi et al., 2015; Hervey-Jumper and Berger, 2016). The use of intraoperative MRI has been demonstrated to aid in achieving maximal safe resection in both tumor surgery (Roder et al., 2014; Coburger et al., 2015; Senft et al., 2011) and epilepsy surgery (Englman et al., 2021; Buchfelder et al., 2000).

The use of intraoperative tractography is not yet widely adopted in clinical practice and its utility remains under investigation. The most common criticisms are the need for high quality images in order for them to be of use in clinical practice, difficulty with registration (Nimsky et al., 2007), the presence of artifacts, variation in agreement on optimal placement of regions of interest, alignment of different imaging modalities (Jacquesson et al., 2019), and time required to acquire and process the images.

Timescale is a particular issue in surgery. For diffusion iMRI reported acquisition times vary from 5 min (Nimsky et al., 2007) to up to 40 min (Yang et al., 2019). These timings exclude post-acquisition processing and tractography generation time for which there is again a wide range of report length from 10 min (Nimsky et al., 2007; D'Andrea et al., 2012) to up to 4 h (Lim et al., 2015). Despite these limitations tractography remains the sole available technique for tracing white matter pathways within the living brain.

There is currently a lack of adequately powered studies that evaluate the use and benefit of intraoperative tractography in terms of surgical outcomes such as EOR and seizure freedom. Systematic reviews and meta-analyses are valuable tools for collating results from these types of studies by analyzing data from reports from multiple centers and surgeons, thereby increasing the confidence we can place in the findings. The aim of this systematic review and meta-analysis was to assess the impact of iMRI tractography on neurosurgical outcomes in both tumor and epilepsy surgeries.

## 2 Method

This review was conducted and the results are presented in accordance with the Preferred Reporting Items for Systematic Reviews and Meta-Analyses (PRISMA) statement (Page et al., 2021). The protocol for this systematic review has been registered in the PROSPERO database (CRD42023427427).

### 2.1 Literature search

A search of five electronic databases, MEDLINE (via Ovid), Embase (via Ovid), the Cochrane Controlled Register of Trials (CENTRAL), SCOPUS, and Web of Science, from their inception, was performed on 2nd February 2021 and repeated to update identified records on 3rd February 2023 using an established protocol (Bramer and Bain, 2017). No filters or limitations were applied in order to identify all relevant papers. Medical Subject Heading (MeSH) terms, keywords, and their synonyms for “diffusion tensor imaging,” “tractography,” “intraoperative,” and “neurosurgery” were combined. The bibliography of eligible articles were manually searched. The full search strategy is available in Supplementary Materials A.

### 2.2 Selection and eligibility criteria

Articles were eligible for inclusion if they were original research papers. The predetermined eligibility criteria for inclusion were: articles reporting use of intraoperative tractography for either epilepsy surgery or resection of a brain or spinal tumor, in adult or pediatric populations, with or without a comparison group that underwent the same neurosurgical procedure in which intraoperative tractography was not performed, and included report of neurosurgical outcome measures such as EOR, mortality rate, seizure freedom, and neurological deficit. All studies that were published in English were included. Non-original articles, such as reviews, letters to the editor, conference proceedings, and commentaries were excluded.

Results of the final searches of all databases were imported to EndNote X9. After removal of duplicates, articles were screened independently by two reviewers (F.Y. & H.A.) in two stages: first reading the title and abstract, second by reading the full text. If reviewers disagreed on the inclusion of an article, consensus was reached through discussion with a third reviewer (J.C).

#### 2.2.1 Data extraction and quality assessment

The following information was extracted from included articles: first author's name, year of publication, title, objective/aim, study design, patient enrolment type (consecutive or selected), patient population (adult, pediatric, or both), sample size of patient population, comparison group, sample size of comparison group, pathology, surgical procedure performed, name of scanner used, field strength of scanner in tesla, DTI parameters [sequence, TE/TR (ms), number of non-collinear directions, *b* values (sec/mm^2^), number of *b*_0_ images, matrix size, slice thickness, bandwidth, number of slices, voxel size], tractography parameters (tracking method, software used, tract visualized, deterministic or probabilistic tractography, default FA threshold, minimum fiber length, region of interest/seeding strategy, time to acquire fiber-tracking images), tractography outcome measures (sensitivity, positive predictive value, negative predictive value), and neurosurgical outcome measures (EOR, immediate outcome, quality of life, recovery time, neurological status, surgery specific outcomes such as seizure freedom and visual field examinations).

The risk of bias in the included studies was assessment using the Methodological Index for Non-Randomized Studies (MINORS) instrument, a tool specifically developed as a quality assessment of surgical studies (Slim et al., 2003).

### 2.3 Data analysis

Summary statistics were generated for study characteristics, DTI protocols, and intraoperative tractography protocols. As a consequence of high variation in study characteristics and disparate reporting of outcomes measures, a statistical analysis using a binary random-effects model using the DerSimonian-Laird method was conducted in R (R Core Team, 2021) using the metafor package (Viechtbauer, 2010) for outcomes that were reported by more than three studies (GTR and STR). Studies in which only some, not all, patients underwent scans that included DTI were excluded from the meta-analysis. Studies that were case reports were also excluded from the meta-analysis.

## 3 Results

### 3.1 Search results and study characteristics

**Figure 2:**
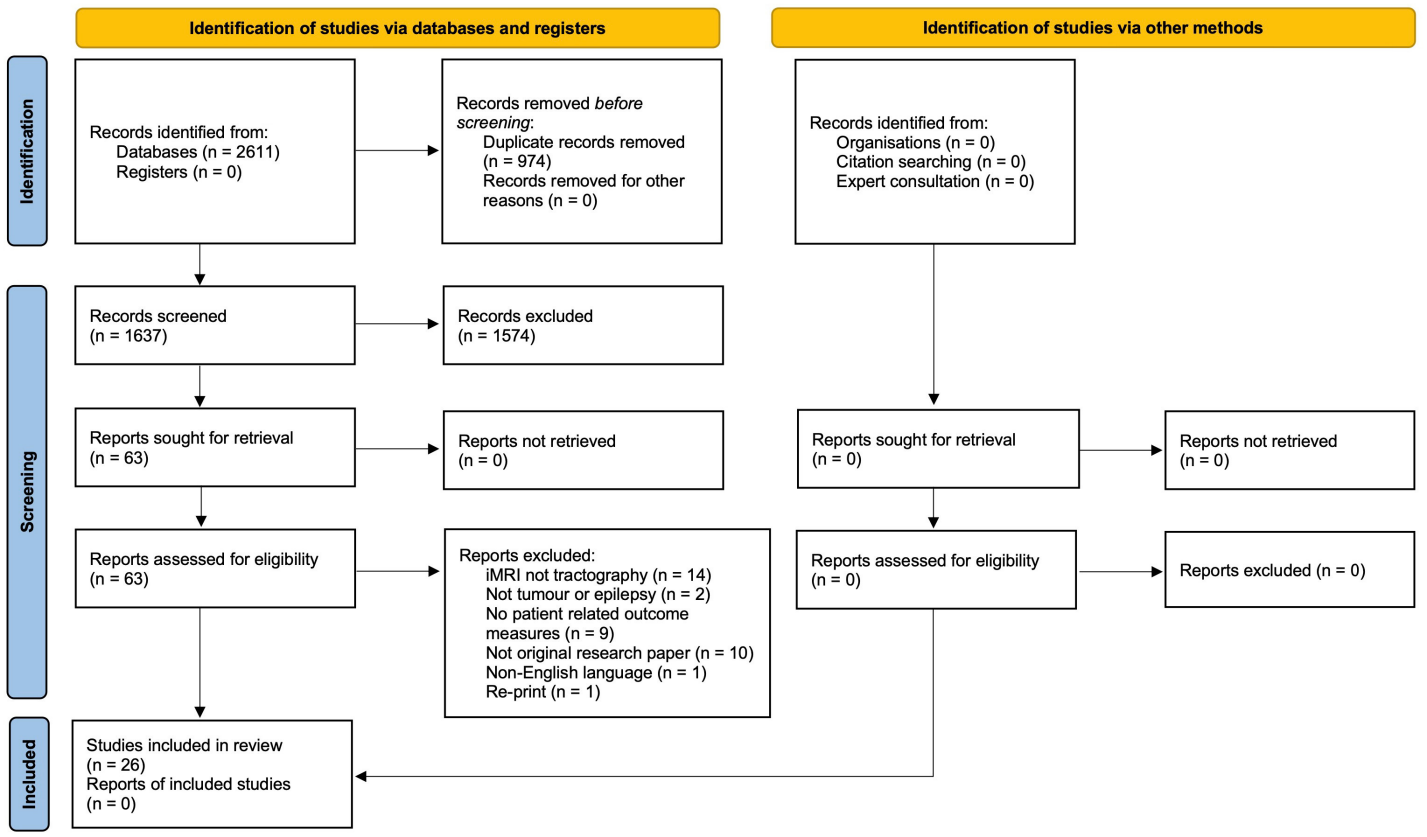
PRISMA 2020 flow diagram for new systematic reviews which included searches of databases, registers and other sources.

A total of 2,611 articles were identified by the search (Figure 2). After the removal of 974 duplicates, 1,637 articles were screened using their title and abstract. Following this initial screening the full text of 63 articles were screened, with 37 articles excluded, leaving 26 articles for inclusion in the review. The reference lists of these 26 articles were then screened for relevant articles that were not identified by the search, however this screening yielded zero results.

Most papers, 92% (24/26), were case series, with the remaining articles, 8% (2/26), case reports (Table 1). All articles were published between 2001 and 2021. Sample sizes ranged from 1 to 142, with 1,043 unique patients across all studies, 28 of which were included in two studies. The age of the patient population is mixed, with 14 studies (54%) consisting of adult patients, and 12 studies (46%) of adult and pediatric patients. Studies of surgery for tumor removal were more common than epilepsy, with 81% (21/26) tumor only, 15% (4/26) epilepsy only, and 4% (1/26) a mix of tumor and epilepsy cases. Only three studies (12%) included comparison groups as part of the study design. The majority of studies did not report on the use of other intraoperative techniques, however ten studies (38%) uploaded the newly acquired intraoperative tractography to their neuronavigation systems (Cui et al., 2014, 2015; D'Andrea et al., 2012, 2017; Li et al., 2016, 2021; Nimsky et al., 2006, 2008, 2005; Sun et al., 2011), one study (4%) employed subcortical stimulation (Bozzao et al., 2010), and one study (4%) used invasive EEG (Sommer et al., 2016).

**Table 1:** Characteristics of included studies.

**First author (year)**	***n* Patients**	**Population**	**Pathology**	**Field strength (tesla)**	**Tract(s) visualized**
Bozzao (2010)	9	Adult	Tumor	1.5	Corticospinal tract
Chen (2009)	48	Pediatric Adult	Epilepsy	1.5	Optic radiation
Cui (2014)	69	Pediatric Adult	Epilepsy	1.5	Corticospinal tract
Cui (2015)	52	Adult	Epilepsy	1.5	Optic radiation
D'Andrea (2011)	1	Adult	Tumor	1.5	Optic radiation
D'Andrea (2012)	18	Adult	Tumor	1.5	Corticospinal tract
D'Andrea (2016)	27	Adult	Tumor	1.5	Arcuate fasciculus
D'Andrea (2017)	142	Adult	Tumor	1.5	Corticospinal tract Arcuate fasciculus Optic radiation
Hajiabadi (2015)	2	Adult	Tumor	1.5	Optic radiation
Hajiabadi (2016)	25	Pediatric Adult	Tumor	1.5	Optic radiation
Javadi (2017)	20	Adult	Tumor	1.5	Corticospinal tract
Leroy (2019)	100	Adult	Tumor	1.5	Corticospinal tract
Li (2016)	12	Pediatric Adult	Tumor	1.5	Corticospinal tract Arcuate fasciculus Optic radiation Medial lemniscus
Li (2021)	54	Adult	Tumor	1.5	Arcuate fasciculus
Maesawa (2009)	100	Pediatric Adult	Tumor	1.5	Unclear
Maesawa (2010)	28	Pediatric Adult	Tumor	1.5	Corticospinal tract
Mamata (2001)	3	Adult	Tumor	0.5	Arcuate fasciculus
Nimsky (2005a)	38	Pediatric Adult	Tumor Epilepsy	1.5	Corticospinal tract Corpus callosum Optic radiation
Nimsky (2005b)	37	Pediatric Adult	Tumor	1.5	Corticospinal tract Corpus callosum
Nimsky (2006)	137	Pediatric Adult	Tumor	1.5	“Major white matter tracts”
Nimsky (2008)	70	Adult	Tumor	1.5	Pyramidal
Ostry (2013)	25	Adult	Tumor	3	Corticospinal tract
Prabhu (2011)	12	Adult	Tumor	1.5	“Fiber structures in the treatment plan”
Sommer (2016)	28	Pediatric Adult	Epilepsy	1.5	Unclear
Sun (2011)	44	Pediatric Adult	Tumor	1.5	Optic radiation
Yuanzheng (2015)	40	Pediatric Adult	Tumor	1.5	Corticospinal tract

### 3.2 Risk of bias analysis

The MINORS tool was applied to assess risk of bias (Table 2). The ideal global score for non-comparative studies is 18 and for comparative studies is 24. The overall mean score was 12.38 (range 9–18). The mean score of non-comparative studies was 11.65 (range 9–14) and comparative studies was 18 (range 18–18).

**Table 2:** Risk of bias analysis conducted using MINORS.

	**Non-comparative study**								**Comparative study**				**Total**
**First author (year)**	**a**	**b**	**c**	**d**	**e**	**f**	**g**	**h**	**i**	**j**	**k**	**l**	
Bozzao (2010)	2	1	2	2	0	2	0	0	–	–	–	–	7
Chen (2009)	2	2	2	2	2	2	2	0	–	–	–	–	14
Cui (2014)	2	1	1	2	0	2	2	0	2	2	2	2	18
Cui (2015)	2	1	1	2	0	2	2	0	2	2	2	2	18
D'Andrea (2011)	2	–	1	2	0	2	2	0	–	–	–	–	9
D'Andrea (2012)	2	1	2	2	0	2	2	0	–	–	–	–	11
D'Andrea (2016)	2	2	1	2	0	2	2	0	–	–	–	–	11
D'Andrea (2017)	2	1	1	2	0	2	2	0	–	–	–	–	10
Hajiabadi (2015)	2	1	1	2	0	2	2	0	–	–	–	–	10
Hajiabadi (2016)	2	2	2	2	0	2	2	0	1	2	2	1	18
Javadi (2017)	2	2	2	2	0	2	2	0	–	–	–	–	12
Leroy (2019)	2	2	2	2	1	2	2	0	–	–	–	–	13
Li (2016)	2	2	2	2	2	2	2	0	–	–	–	–	14
Li (2021)	2	1	2	2	2	2	2	0	–	–	–	–	13
Maesawa (2009)	2	2	2	2	0	2	2	0	–	–	–	–	12
Maesawa (2010)	2	1	2	2	0	2	2	0	–	–	–	–	11
Mamata (2011)	2	1	2	2	0	2	2	0	–	–	–	–	11
Nimsky (2005a)	2	0	2	2	0	2	2	0	–	–	–	–	10
Nimsky (2005b)	2	0	2	2	0	2	2	0	–	–	–	–	10
Nimsky (2006)	2	2	2	2	0	2	2	0	–	–	–	–	12
Nimsky (2008)	2	0	2	2	0	2	2	0	–	–	–	–	10
Ostry (2013)	2	2	2	2	2	2	2	0	–	–	–	–	14
Prabhu (2011)	2	1	1	2	0	2	2	0	–	–	–	–	10
Sommer (2016)	2	1	1	2	0	2	2	0	–	–	–	–	10
Sun (2011)	2	2	2	2	2	2	2	0	–	–	–	–	14
Yuanzheng (2015)	2	2	2	2	2	2	2	0	–	–	–	–	14

Key: (a) Clearly stated aim, (b) Inclusion of consecutive patients, (c) Prospective collection of data (d) Endpoints appropriate to the aim, (e) Unbiased assessment of study endpoint, (f) Follow-up period appropriate to the aim, (g) Loss of follow up < 5%, (h) Prospective calculation of study size, (i) Adequate control group, (j) Contemporary groups, (k) Baseline equivalance of groups, (l) Adequate statistical analysis.

### 3.3 Use of intraoperative DTI

The majority of studies used a 1.5 tesla MRI field strength (92%, 24/26) (Table 3). The remaining studies used either a 3T (4%, 1/26) or 0.5T scanner (4%, 1/26). The optic radiation was visualized in 27% (7/26) of studies, the corticospinal tract in 50% (13/26) of studies, the arcuate fasciculus in 19% (5/26) of studies, the corpus callosum in 8% (2/26), the medial lemniscus in 4% (1/26) of studies. In 19% (5/26) of studies is was unclear which tracts were visualized.

**Table 3:** Characteristics of imaging protocols in included studies.

**First author (year)**	**Scanner**	**Field strength (tesla)**	**Sequence**	**TE/TR (ms)**	**# Non-collinear directions**	***b* value (sec/mm^2^)**	**# of *b*_0_ images**	**Voxel-size (mm)**
Bozzao (2010)	Siemens Sonata	1.5	Echoplanar	86/9,200	12	1,000	1	1.9 × 1.9 × 1.9
Chen (2009)	Siemens Sonata	1.5	Single-shot spin-echo diffusion-weighted echo planar	86/9,200	6	1,000	1	1.9 × 1.9 × 1.9
Cui (2014)	Siemens Espree	1.5	Single-shot spin-echo diffusion weighted echo planar	147/9,400	12	1,000	1	1.9 × 1.9 × 3
Cui (2015)	Siemens Espree	1.5	Single-short spin-echo diffusion weighted echo planar	147/9,400	12	1,000	1	1.9 × 1.9 × 3
D'Andrea (2011)	Siemens Sonata	1.5	Echoplanar	86/9,200	6	1,000	1	1.9 × 1.9 1.9
D'Andrea (2012)	Siemens Magnetom Sonata	1.5	Echoplanar	92/9,400	12	1,000	1	1.8 × 1.8 × 1.9
D'Andrea (2016)	Siemens Sonata	1.5	Echoplanar	86/9,200	6	10,00	1	1.9 × 1.9 × 1.9
D'Andrea (2017)	Siemens Sonata	1.5	Echoplanar	86/9,200	12	1,000	1	1.9 × 1.9 1.9
Hajiabadi (2015)	Siemens Magnetom Espree	1.5	Rapid gradient echo	–	20	–	–	–
Hajiabadi (2016)	Siemens Magnetom Sonata	1.5	Rapid acquisition gradient-echo	–	20	1,000	–	–
Javadi (2017)	Siemens Magnetom Sonata	1.5	–	88/10,000	12	1,000	–	–
Leroy (2019)	General Electric	1.5	–	–	–	1,000	1	–
Li (2016)	Siemens Magnetom Sonata	1.5	Single-shot spin-echo diffusion-weighted echoplanar	147/94,00	12	1,000	1	–
Li (2021)	Siemens Espree	1.5	Single-shot spin-echo diffusion-weighted echoplanar	147/9,400	12	1,000	1	1.9 × 1.9 × 3
Maesawa (2009)	Siemens Magnetom Symphony	1.5	Single-shot spin-echo diffusion-weighted echoplanar	99/7,500	–	–	–	–
Maesawa (2010)	Siemens Magnetom Symphony	1.5	Single-shot spin-echo diffusion-weighted echoplanar	99/7,500	12	1,000	1	–
Mamata (2001)	SIGNA SP	0.5		163/230	6	500		1.7 × 1.7 × 7
Nimsky (2005a)	Siemens Magnetom Sonata Maestro Class	1.5	Single-shot spin-echo diffusion-weighted echo planar	86/9,200	6	1,000	1	1.9 × 1.9 × 1.9
Nimsky (2005b)	Siemens Magnetom Sonata Maestro Class	1.5	Single-shot spin-echo diffusion-weighed echo planar	86/9,200	6	1,000	1	1.9 × 1.9 × 1.9
Nimsky (2006)	Siemens Magnetom Sonata Maestro Class	1.5	–	86/9,200	–	–	–	–
Nimsky (2008)	Siemens Magnetom Sonata Maestro Class	1.5	Single-shot spin-echo diffusion-weighted echo planar	86/9,200	6	1,000	1	1.9 × 1.9 × 1.9
Ostry (2013)	General Electric Signa HD	3	–	–	25	1,000	1	1.8 × 1.8 × 5
Prabhu (2011)	Siemens Espree	1.5	Spin-echo echoplanar with parallel imaging	100/6,700	20	750	1	1.9 × 1.9 × 3
Sommer (2016)	Siemens Magnetom Sonata Maestro Class	1.5	Spin-echo echoplanar with parallel imaging	–	–	–	–	–
Sun (2011)	Siemens Espree	1.5	Single-shot spin-echo diffusion-weighted echoplanar	147/9,400	12	1,000	1	1.9 × 1.9 × 3
Yuanzheng (2015)	Siemens Espree	1.5	Single-shot spin-echo diffusion-weighted echoplanar	147/9,400	12	1,000	1	1.9 × 1.9 × 3

### 3.4 Use of intraoperative tractography

Intraoperative tractography was conducted in all studies (Table 4). All studies that reported the software program employed for tractography (69%, 18/26) used BrainLab (Feldkirchen, Germany) software. iPlan 2.6 was the most commonly used (35%, 9/26), followed by iPlan 3.0 and DTI Task Card Version 1.6 (12%, 3/26), and iPlan 2.5 (8%, 2/26), with one study not reporting which BrainLab package was used (4%). Reported default FA thresholds ranged from 0.01 to 0.3, with 0.15 being the most common (19%, 5/26). 10 studies did not report a threshold (38%). Reported minimum fiber lengths ranged from 5 to 100 mm. The majority of studies did not report a measurement (56%, 15/26), of those who did the most commonly reported length was 50 mm (27%, 7/26). Most studies also did not report image processing time (56%, 15/26). Of studies that did, processing time ranged from 2 to 27.5 min.

**Table 4:** Characteristics of intraoperative tractography in included studies.

**First author (year)**	**Tract(s) visualized**	**Software used**	**Default FA threshold**	**Minimum fiber length (mm)**	**Processing time (mins)**	**ROI strategy**
Bozzao (2010)	Corticospinal tract	DTI task card version 1.6 (BrainLab, Feldkirchen, Germany)	0.2	–	2–3	30 voxel ROIs positioned in posterior limb of internal capsule.
Chen (2009)	Optic radiation	iPlan 2.5 (BrainLab, Feldkirchen, Germany)	0.15	50	15	Multi-VOI algorithm. For Meyer's Loop: VOI 1 on lateral geniculate body, VOI 2 on level of lower lip of visual occipital cortex. For optic radiation VOI 1 on lateral geniculate nucleus and VOI 2 on level of middle and upper lip of visual occipital cortex.
Cui (2014)	Corticospinal tract	–	–	–	–	First ROI on subcortical WM in pre-central gyrus, second ROI on cerebral peduncle. For sensory tract, first ROI on subcortical WM in post-central gyrus and second ROI on cerebral peduncle.
Cui (2015)	Optic radiation	Fiber-tracking module of iPlan 2.6 (Brainlab, Feldkirchen, Germany)	0.15	50	10	Multi-VOI algorithm for fiber tracking of optic radiation. For Meyer's Loop first VOI placed on the lateral geniculate body. Second VOI placed to cover the lower lip of the visual occipital cortex. For dorsal bundle of optic radiation, first VOI placed on lateral geniculate body, second VOI placed to cover the middle and upper lip of the visual occipital cortex.
D'Andrea (2011)	Optic radiation	DTI task card version 1.6 (BrainLab, Feldkirchen, Germany)	0.2	–	2–3	ROI positioned in the lateral geniculate ganglion.
D'Andrea (2016)	Arcuate fasciculus	DTI task card version 1.6 (BrainLab, Feldkirchen, Germany)	0.17	–	15	ROI positioned in lateral region of corticospinal tract along the cranial limit of the splenium of the corpus callosum.
D'Andrea (2017)	Corticospinal tract Arcuate fasciculus Optic radiation	iPlan 2.6 (BrainLab, Feldkirchen, Germany)	0.17	–	15	Internal capsule for corticospinal tract, geniculate ganglion for optic radiation, in right-handed patients with left lesions the ROI encompassed horizontal fibers lateral to coronal radiata and medial to cortex of posterior part of ventrolateral frontal lobe.
D'Andrea (2012)	Corticospinal tract	iPlan 2.6 (BrainLab, Feldkirchen, Germany)	0.17	–	15	Posterior arm of the internal capsule and precentral gyrus for corticospinal tract. Initiated in retrograde and orthograde directions according to direction of principal eigenvector in each voxel of the ROI.
Hajiabadi (2015)	Optic radiation	–	0.15	21	–	Three standard ROIs: chiasma, lateral to the trigone, and occipital lobe.
Hajiabadi (2016)	Optic radiation	BrainLab (Feldkirchen, Germany)	0.01	5	–	Seed regions at optic chiasm, occipital cortex, and deep regions between superior and middle temporal gyri lateral to trigone.
Javadi (2017)	Corticospinal tract	iPlan 3.0 (BrainLab, Feldkirchen, Germany)	0.1–0.3	80–100	–	Selection of ROIs at the precentral gyrus and ipsilateral cerebral peduncle.
Leroy (2019)	Corticospinal tract	–	–	–	–	–
Li (2016)	Corticospinal tract Medical lemniscus Arcuate fasciculus Optic radiation	iPlan 3.0 (BrainLab, Feldkirchen, Germany)	–	–	–	–
Li (2021)	Arcuate fasciculus	iPlan 3.0 (BrainLab, Feldkirchen, Germany)	0.15	50	–	Three ROIs. First ROI placed on left inferior frontal gyrus, second ROI placed on lateral part of the corona radiata, third ROI placed on posterior part of left superior temporal gyrus.
Maesawa (2010)	Corticospinal tract	iPlan 2.6 (BrainLab, Feldkirchen, Germany)	0.3	–	< 10	For corticospinal tract: WM fibers emerging from cerebral peduncle ipsi-lateral to lesion were tracked to pre-central gyrus.
Maesawa (2009)	–	–	–	–	–	–
Mamata (2001)	Arcuate fasciculus	–	–	–	–	–
Nimsky (2008)	Corticospinal tract	Imagefusion module, iPlan 2.5 (BrainLab, Feldkirchen, Germany)	–	–	2	–
Nimsky (2006)	–	–	–	–	–	–
Nimsky (2005a)	Corticospinal tract Corpus callosum Optic radiation	–	–	–	–	–
Nimsky (2005b)	Corticospinal tract Corpus callosum	–	0.3	–	27.5	Corticospinal tract: two-seed-region approach, one seed region on precentral gyrus, second seed region in area of the internal capsule. Corpus Callosum: whole corpus callosum in midsagittal plane used.
Ostry (2013)	Corticospinal tract	iPlan 2.6 (BrainLab, Feldkirchen, Germany)	0.1–0.15	50	–	Three ROIs for corticospinal tract (cerebral peduncle, internal capsule, and subcortical area below precentral gyrus). Clear contaminating fibers excluded manually.
Prabhu (2011)	–	iPlan 2.6 (BrainLab, Feldkirchen, Germany)	–	–	–	Initial seed ROI in the selected image set using the DTI ROI.
Sommer (2016)	–	iPlan 2.6 (BrainLab, Feldkirchen, Germany)	50	–	–	–
Sun (2011)	Optic radiation	iPlan 2.6 (BrainLab, Feldkirchen, Germany)	0.15	50	10	Multi-VOI algorithm for optic radiation. For Meyer's Loop, first VOI placed on lateral geniculate body. Second VOI placed to cover lower lip of visual occipital cortex. For dorsal bundle of optic radiation, first VOI placed on lateral geniculate body, second VOI placed to cover middle and upper lip of the visual occipital cortex.
Yuanzheng (2015)	Corticospinal tract	Fiber-tracking module of iPlan 2.6 (BrainLab, Feldkirchen, Germany)	0.15	50	–	Two VOI approach. First VOI positioned immediately below precentral gyrus. Second VOI stationed at cerebral peduncle.

All anatomical location names are as reported in each study. Key: Region of Interest (ROI), Volume of Interest (VOI).

### 3.5 Surgical outcomes: GTR

**Figure 3:**
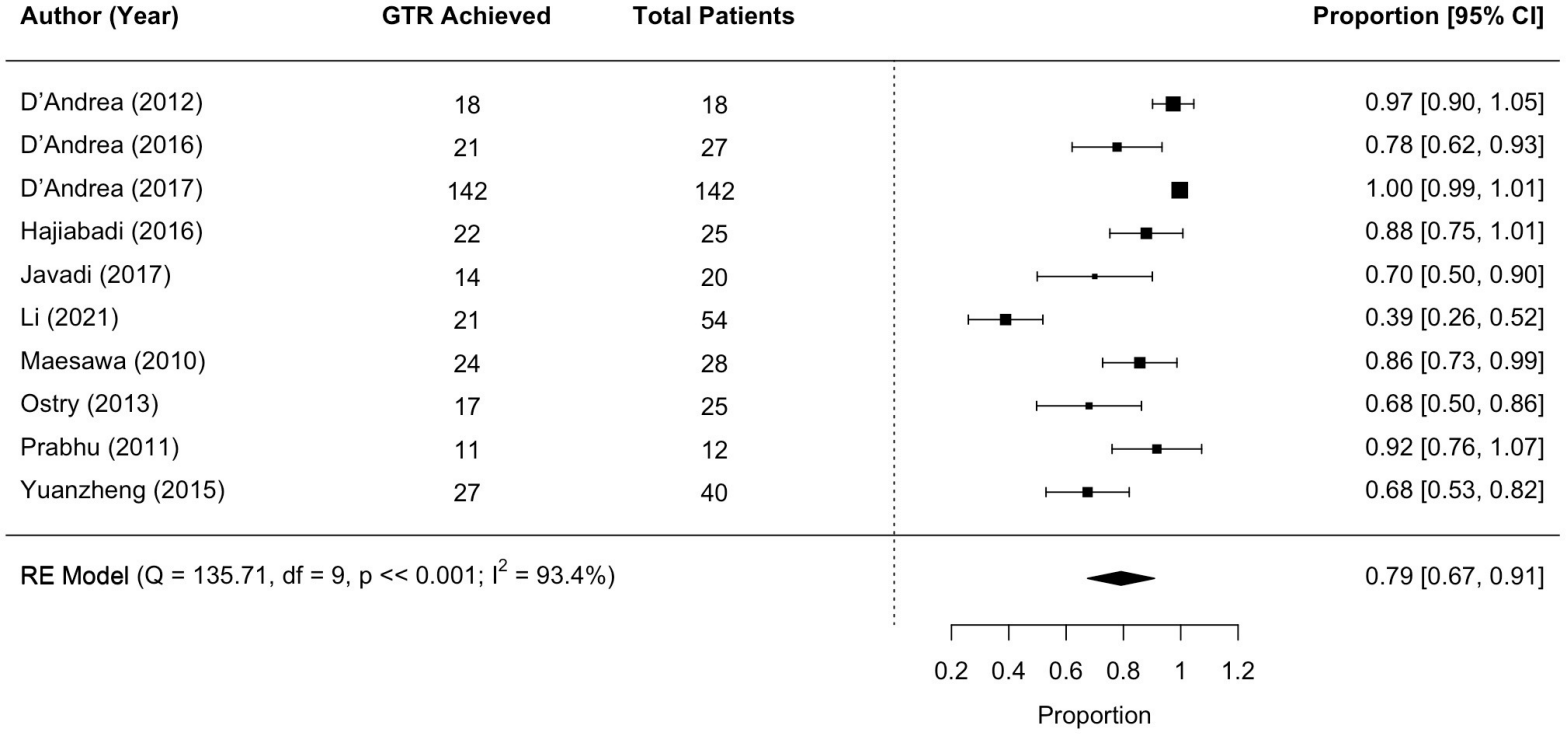
Forest plot of Gross Total Resection (GTR) rates.

Eleven studies met the meta-analysis inclusion criteria, with 392 unique tumor patients. The gross total resection (GTR) rate was 79% (range 67–91; *I*^2^ = 93.4%, P heterogeneity ≪0.001; Figure 3).

### 3.6 Surgical outcomes: STR

**Figure 4:**
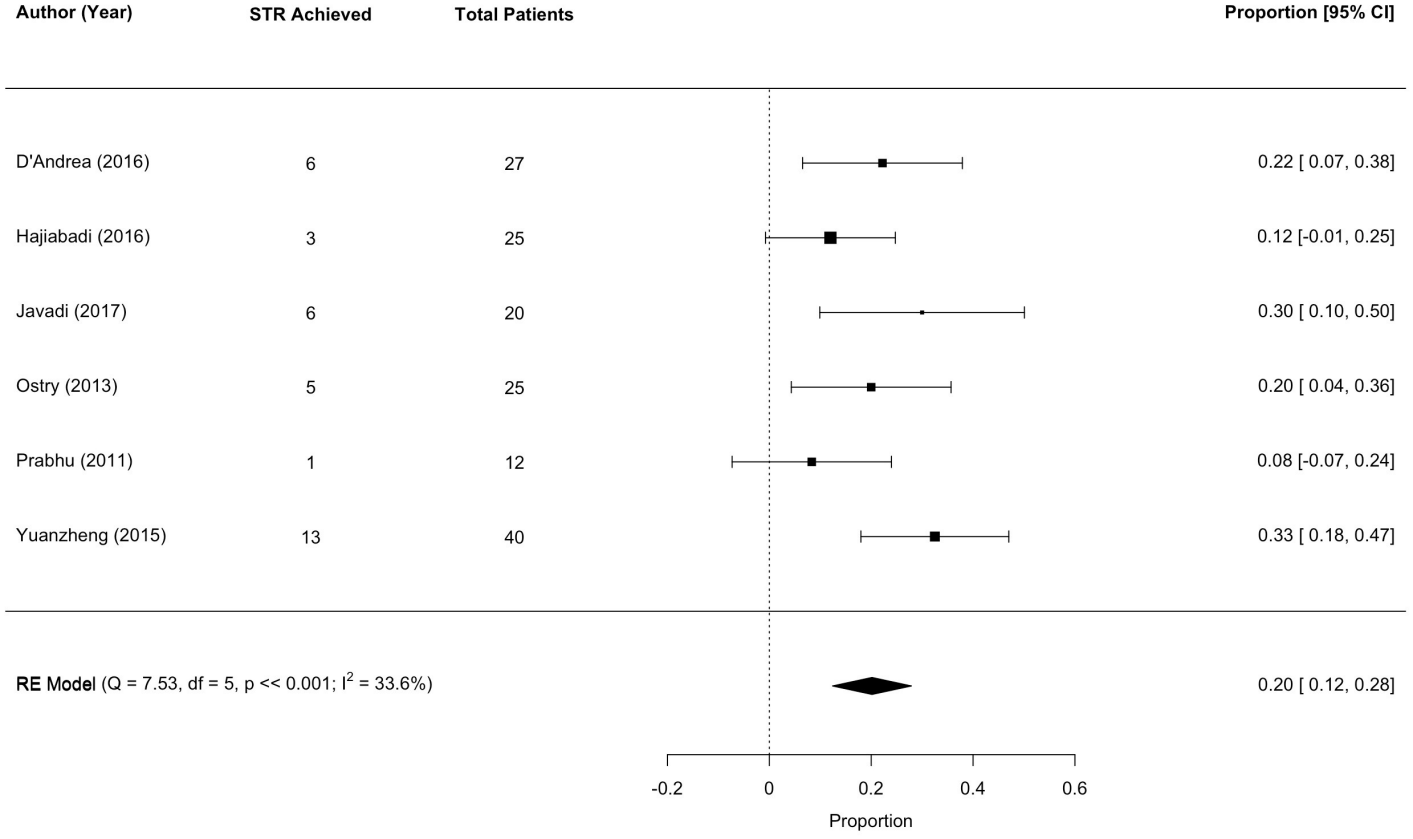
Forest plot of Sub-Total Resection (STR) rates.

Six studies met the meta-analysis inclusion criteria, with 149 unique tumor patients. The sub-total resection (STR) rate was 20% (range 12–28; *I*^2^ = 33.6%, *P* heterogenity ≪0.001; Figure 4).

### 3.7 Surgical outcomes: tractography

Only one study reported outcome measures relating to the intraoperative tractography, finding 100% sensitivity, 78% positive predictive value, and 100% negative predictive value (Javadi et al., 2017).

### 3.8 Surgical outcomes: brain tumors

Eleven studies reported neurological deficits as an outcome measure (Table 5).

**Table 5:** Surgical outcomes in included studies of brain tumors.

**First author (year)**	***n* Patients**	**Pathology and procedure**	**Report of neurological deficit**
D'Andrea (2016)	27 adults	Tumor, resection	Normal neurologic status in 22/27 (81.5%), improvement from preoperative symptoms in 20/27 (74%)
D'Andrea (2017)	142 adults	Tumor close to one or more white matter tract(s), resection	Normal neurologic status in 81.5%, improvement from preoperative symptoms in 74%
D'Andrea (2012)	18 adults	Tumor involving motor cortext and/or corticospinal tract, resection	Immediate outcome improved in 10/18 (55%), Unchanged in 6/18 (33%), mild paresis in 1/18 (0.05%), monoparesis in 1/18 (5%)
Javadi (2017)	20 adults	Supratentorial gliomas adjacent to corticospinal tract, resection	Significant improvement with no new permanent neurological deficits in all of the patients
Li (2016)	12 adult and pediatric	Supratentorial cavernomas, resection	Some or all presenting signs and symptoms were improved or resolved in four cases but were unchanged in seven patients
Mamata (2001)	3 adults	Tumor, resection (1 under GA, 2 awake craniotomy)	No postoperative neurological deficits.
Nimsky (2008)	70 adults	Tumors adjacent to the pyramidal tract, resection	3/70 (4.2%) had new permanent neurological deficit
Nimsky (2005a)	38 adult and pediatric	Tumor and epilepsy, 35 craniotomies and 3 burr hole procedures	No patient developed new neurologic deficits due to tumor resection
Nimsky (2005b)	37 adult and pediatric	Supratentorial tumors, resection	1/37 (2.7%) new postoperative neurological deficit
Prabhu (2011)	12 adults	Tumor, resection	New/worsening neurological deficits were observed in 7 (58%) of patients, in 2 (17%) a persistent neurological deficit was noted at 3 months
Yuanzheng (2015)	40 adult and pediatric	Low and high grade gliomas adjacent to corticospinal tract, resection	Three (7.5%) patients had permanent aggravated deficits but could live independently at 3–12 month follow up

Only one study reports the use of cognitive assessment measures following surgery, finding the median Karnofsky Performance Scale score in 100 adult patients undergoing resection for low and high grade gliomas at discharge, 3 months, 6 months, 9 months, and 1 year post surgery to be 90, indicating normal activity (Leroy et al., 2019).

Three studies employed quality of life measures post operatively, with two reporting “Excellent” in over 85% of patients [85.1% (D'Andrea et al., 2017) and 88% (D'Andrea et al., 2012)] and another finding no deficits affecting normal quality of life in 85.1% of patients with 14.8% having a moderate deficit (D'Andrea et al., 2016).

Assessments of motor function were used in five studies. Bozzao et al. (2010) report that motor function was preserved in all nine adult patients with one showing initial transient weakness that resolved within 1 month. In a study of 142 adult patients motor function was preserved in all but three who showed transient weakness on the contralateral side that improved between 1 and 3 months post-surgery (D'Andrea et al., 2017).

Similarly, in 28 adult and pediatric patients undergoing resection of gliomas near the corticospinal tract, 12 (42.8%) had transient deterioration of motor function, but improved to baseline between 1 day and 2 weeks following surgery, with 3.5% developing permanent paresis (Maesawa et al., 2010). In a further 25 adult patients also undergoing resection of tumors infiltrating the corticospinal tract, motor deficits were noted in eight (32%), with seven going on to regain function to preoperative status within 1 month (Ostrý et al., 2013). Yuanzheng and colleagues report no changes or improvement in motor deficits in 24 of 40 pediatric and adult patients (60%), with aggravated new deficits in 14 (40%) 1 week post surgery, and three developing permanent deficits after 3–12 months but with preserved ability to live independently (Yuanzheng et al., 2015).

In two studies in which patients underwent resection of a tumor adjacent to the optic radiation visual field assessments are reported. Hajiabadi et al. (2015) report both visual field and acuity measurements improved significantly 3 months post surgery in two adult patients. Similarly, in 44 adult and pediatric patients there was no change in 36 (81.8%), improved scores in five (11.4%), and aggravated defects in three (6.8%) (Sun et al., 2011).

Two studies report speech assessment measures. In 54 adult patients undergoing tumor resection adjacent to the arcuate fasciculus, 15 patients (27.8%) experienced worsened language function in comparison to preoperative function, with mean Western Aphasia Battery score decreasing from 90 to 66 signifying a poor outcome (Li et al., 2021). However, in a study of 25 adult patients undergoing resection of tumors infiltrating the corticospinal tract, speech disorders developed in the dominant parietal lobe in four patients (16%), with three fully recovering within 3 months (Ostrý et al., 2013).

### 3.9 Surgical outcomes: epilepsy

Seizure freedom was employed as an outcome measure in one study. In a study of 27 adult and pediatric patients with frontal lobe epilepsy undergoing corticotomies or extended lesionectomies there was excellent seizure control in 18/28 (65%, Engel Class 1A) of patients and a poor outcome in 6/28 (21%) (Sommer et al., 2016).

One study reported measures of visual field defects in epilepsy surgery. In 48 adult and pediatric patients with pharmaco-resistant temporal lobe epilepsy undergoing temporal lobectomy there was a significant correlation between field loss, measured by visual field defect grade, and injury fraction of the optic radiation/Meyer's loop (*p* = < 0.001) (Chen et al., 2009).

### 3.10 Comparison groups

Only three studies incorporated comparison groups of patients who underwent surgery without the aid of intraoperative tractography (11%). Cui et al. (2014) compared 31 pediatric and adult patients undergoing epilepsy surgery aided by tractography to 38 patients undergoing surgery not aided by tractography and found, at two year follow up, that more patients in the iMRI group had a good outcome as rated on Engel's Classifcation, a seizure outcome scale, though the result was not statistically significant (71 vs. 55.3%, *p* = 0.181). New post-operative neurological deficits 1 week after the operation were present in 50% of the control group compared to 25.8% in the iMRI group (Cui et al., 2014). Within one year of the surgery 18.4% of the control group and 9.7% of the iMRI group did not recover to their pre-operative strength, and iMRI patients demonstrated significantly less hemiparesis 1 week post surgery (*p* = 0.043) (Cui et al., 2014).

Cui et al. (2015) compared 20 adult patients undergoing epilepsy surgery aided by tractography to 32 patients undergoing surgery not aided by tractography and found, in contrast to the previous study, that 6 months after surgery the control group outperformed the iMRI group on the Engel's Classification, though again the result was not statistically significant (*p* = 0.537). Hajiabadi et al. (2016) compared 25 pediatric and adult patients undergoing tumor resection aided by intraoperative tractography with six control patients who had normal vision and underwent other surgeries for other pathologies and found all but one patient had improved visual status after the surgery. Visual improvement after tumor removal was significantly correlated with distance between the optic tract and the tumor visualized in the intraoperative tractography at both 1 week and 3 months (*p* < 0.01). Additionally there was a statistically significant correlation between detection of chiasm-crossing fibers in tractography 1 week after resection and visual improvement seen 3 months after the operation (*p* = 0.002).

## 4 Discussion

This systematic review and meta-analysis demonstrates that intraoperative tractography has the potential to improve surgical outcomes for patients with brain tumors and epilepsy. Identified studies showed a moderate risk of bias, however processing time, an aforementioned concern in the clinical use of intraoperative tractography, ranged from 2 to 27.5 min. The meta-analysis found a good rate of GTR (79%), though rates of GTR and STR are related to tumor grade and not all studies reported the type or grading of tumors. In all studies intraoperative tractography was conducted using BrainLab software which employs a version of the deterministic FACT algorithm (Mori et al., 1999) and the diffusion tensor fiber orientation model (BrainLab, 2012). Therefore, the resulting visualized tracts are not representative of the full potential of intraoperative tractography if technical limitations such as long processing times could be overcome to utilize more advanced techniques.

In tumor surgeries only three studies reported minor aggravated neurological deficits (Yuanzheng et al., 2015; Sun et al., 2011; Li et al., 2021) with the other 19 studies either reporting no change in symptoms or an improvement in cognitive function, quality of life, motor function, visual fields, and speech.

In epilepsy surgeries only one study reported a poor outcome in 22% of their patients, classified as class 3 or 4 on the Engel Epilepsy Surgery Outcome Scale, with 71% of patients classified as class 1 (seizure free) and 7% as class 2 (rare seizures) (Sommer et al., 2016). While seizure freedom is reported in several studies, the utility of intraoperative tractography in epilepsy surgery is ultimately to improve functional prognosis by preserving WM tracts. Statistically significant correlations between fiber tracking estimation and visual field defect outcomes along with correlations between the defect and the injury visualized on fiber tracking were found in one study (Chen et al., 2009). This is the only non-comparative study in which intraoperative tractography can be linked to a direct impact on neurological outcomes. The other non-comparative studies do not directly relate neurological outcomes to the use of intraoperative tractography in a way that can be disentangled from the outcome of the surgery.

Two of the three studies with comparison groups undertaken in epilepsy surgery did not find a statistically significant difference in Engel Classification outcomes (Cui et al., 2014, 2015). This outcome may have been affected by selection bias as both studies retrospectively selected patients (Table 2). The one study in tumor surgery with a comparison group demonstrated a statistically significant correlation between the detection of chiasm crossing fibers and visual improvement, though the comparison group only contained six patients who were undergoing other surgeries for other pathologies undermining the strength of the comparison (Hajiabadi et al., 2016).

Small sample sizes were the most common limitation across identified studies, with 20/26 (77%) studies including under 50 patients. None of the studies were gold standard randomized controlled trials, although conducting this study design in surgical research is acknowledged to be difficult and in some cases unethical (McCulloch et al., 2002). However, 10/26 (38%) of included studies were prospective studies of consecutive patients, reducing the possible selection bias in which patients with certain pathologies undergoing operations with particular approaches are pre-selected to validate intraoperative tractography's use. The wide variation in imaging techniques and tracts visualized is a further limitation making comparison difficult, and intraoperative time constraints impact the quality of tract reconstructions. Furthermore, in tumor cases the extent of resection and incidence of neurological deficit is influenced by factors outside of the use of intraoperative imaging including the grade and eloquence of the tumor, level of infiltration of the tract, and possible surgical injury.

Taken together, evidence for the effect of intraoperative tractography on neurosurgical outcomes is mixed. Intraoperative tractography can be used to identify critical functional pathways, to guide surgical resection, and aid in the achievement of safe maximum total resection, in a time frame that is feasible within surgery without harming the patient. This echoes findings of a systematic review and meta-analysis of iMRI in awake microsurgical resection in gliomas (Tuleasca et al., 2021). However, as most studies did not include a comparison group that did not utilize intraoperative tractography, we cannot be sure that intraoperative tractography specifically, and not for instance iMRI alone, helped achieve the results reported.

The ultimate utility of intraoperative tractography will relate to the tract being reconstructed. While visualization of the corticospinal tract and optic radiation can aid in the preservation of motor function and visual fields, functions that are supported by complex networks of structures, such as language, cannot be preserved this way and would still require awake surgery. However, intraoperative tractography could still provide some assistance here in the context of visualizing the arcuate fasciculus in pediatric neurosurgery, a population for which awake surgery is particularly challenging. Additionally, while time and access to expertise are already acknowledged barriers to the wider use of intraoperative tractography, access to iMRI facilities is itself a significant economic and logistical difficulty preventing adoption in many centers.

More rigorous comparative studies designed to separate the effect of the surgery itself and iMRI from the effect of the addition of intraoperative tractography into the surgical protocol are needed to determine any definitive benefit, and ascertain whether certain patient populations, pathologies, and operative approaches benefit more than others. Variations in intraoperative tractography protocols should also be explored to assess if there is a correlation between the quality of tract reconstruction and patient outcomes.

In conclusion, this systematic review and meta-analysis provides a comprehensive overview of the current use of intraoperative tractography in tumor and epilepsy surgery. The results suggest that intraoperative tractography can be a valuable tool in improving neurosurgical outcomes and reducing the risk of postoperative neurological deficits. However, there is a need for comparative studies that determine whether improvements in neurological outcomes and the achievement of maximum EOR can be directly attributed to the inclusion of intraoperative tractography in order to assess its added value. While structural iMRI is currently utilized widely in clinical practice, diffusion iMRI and intraoperative tractography are not as a result of the technical challenges, and further research is required to determine the optimal use in clinical practice.

## Data Availability

The original contributions presented in the study are included in the article/Supplementary material, further inquiries can be directed to the corresponding author/s.
