# Supplementary material for: The use of intraoperative tractography in brain tumor and epilepsy surgery: a systematic review and meta-analysis
Source: Front Neuroimaging. 2025 Jun 17;4:1563996. doi: 10.3389/fnimg.2025.1563996 (PMC12209374; doi:10.3389/fnimg.2025.1563996)
Supplement: Supplementary file 1 [file Data_Sheet_1.pdf]

Supplementary Material

1 FULL SEARCH STRATEGY

1.1 MEDLINE and Embase

1. exp diffusion tensor imaging
2. exp diffusion magnetic imaging
3. diffusion tensor imag*
4. diffusion imag*
5. diffusion tractogra*
6. DTI
7. tractogra*
8. diffusion tensor MRI
9. diffusion tensor magnetic resonance imag*
10. 1 or 2 or 3 or 4 or 5 or 6 or 7 or 8 or 9
11. exp intraoperative period
12. intraoperative
13. intra-operative
14. intrasurgical
15. intra-surgical
16. intraoperative MRI
17. intra-operative MRI
18. intraoperative magnetic resonance imag*
19. intra-operative magnetic resonance imag*
20. iMRI
21. ioMRI
22. 11 or 12 or 13 or 14 or 15 or 16 or 17 or 18 or 19 or 20 or 21
23. exp neurosurgery
24. neurosurg*
25. during neurosurg*
26. 23 or 24 or 35
27. 10 and 22 and 26

1.2 CENTRAL

1. [MeSH]: Diffusion Tensor Imaging
2. diffusion tensor imag*
3. diffusion imag*
4. DTI
5. tractogra*
6. diffusion tractogra*
7. diffusion tensor MRI
8. diffusion tensor magnetic resonance imag*
9. 1 OR 2 OR 3 OR 4 OR 5 OR 6 OR 7 OR 8

10. intraoperative
11. intra-operative
12. intrasurgical
13. intra-surgical
14. intraoperative MRI
15. intra-operative MRI
16.
17. intraoperative magnetic resonance imag*
18. intra-operative magnetic resonance imag*
19. iMRI
20. ioMRI
21. 10 OR 11 OR 12 OR 13 OR 14 OR 15 OR 16 OR 17 OR 18 OR 19
22. [MeSH]: Neurosurgery
23. neurosurg*
24. during neurosurg*
25. 21 OR 22 OR 23
26. 9 AND 20 AND 24

1.3 SCOPUS

(TITLE-ABS-KEY(diffusion tensor imag*) OR (diffusion imag*) OR (DTI) OR (tractogra*) OR (diffusion tractogra*) OR (TBSS) OR (diffusion tensor MRI) OR (diffusion tensor magnetic resonance imag*)) AND (TITLE-ABS-KEY(intraoperative OR intra-operative OR intrasurgical OR intra-surgical OR intraoperative MRI OR intra-operative OR intraoperative magnetic resonance imag* OR intra-operative magnetic resonance imag* or iMRI or ioMRI)) AND (TITLE-ABS-KEY(neurosurg* OR during neurosurg*))

1.4 Web of Science

TS=(diffusion tensor imag*) OR TS=(diffusion imag*) OR TS=(DTI) OR TS=(tractogra*) OR TS=(diffusion tractogra*) OR TS=(TBSS) OR TS=(diffusion tensor MRI) OR TS=(diffusion tensor magnetic resonanc[illegible] AND TS=(intraoperative) OR TS=(intra-operative) OR TS=(intrasurgical) OR TS=(intra-surgical) OR TS=(intraoperative MRI) OR TS=(intra-operative MRI) OR TS=(intraoperative magnetic resonance imag*) OR TS=(intra-operative magnetic resonance imag*) OR TS=(iMRI) OR TS=(ioMRI) AND TS=(neurosurg*) OR TS=(during neurosurg*)
